# Supplementary material for: Experience shapes activity dynamics and stimulus coding of VIP inhibitory cells
Source: eLife. 2020 Feb 26;9:e50340. doi: 10.7554/eLife.50340 (PMC7043888; doi:10.7554/eLife.50340)
Supplement: Transparent reporting form [file elife-50340-transrepform.docx]

***eLife’s* transparent reporting form**

We encourage authors to provide detailed information *within their submission* to facilitate the interpretation and replication of experiments. Authors can upload supporting documentation to indicate the use of appropriate reporting guidelines for health-related research (see [EQUATOR Network](http://www.equator-network.org/%20)), life science research (see the [BioSharing Information Resource](https://biosharing.org/)), or the [ARRIVE guidelines](http://www.plosbiology.org/article/info:doi/10.1371/journal.pbio.1000412) for reporting work involving animal research. Where applicable, authors should refer to any relevant reporting standards documents in this form.

If you have any questions, please consult our Journal Policies and/or contact us: [editorial@elifesciences.org](mailto:editorial@elifesciences.org).

**Sample-size estimation**

- You should state whether an appropriate sample size was computed when the study was being designed
- You should state the statistical method of sample size computation and any required assumptions
- If no explicit power analysis was used, you should describe how you decided what sample (replicate) size (number) to use

Please outline where this information can be found within the submission (e.g., sections or figure legends), or explain why this information doesn’t apply to your submission:

There was no explicit power analysis used for estimating sample size during study design. Previous experiments using our imaging protocols (e.g. de Vries et al., 2020, Nature Neuroscience) indicated that n = 6 mice per condition was sufficient to capture variability in cortical activity.

**Replicates**

- You should report how often each experiment was performed
- You should include a definition of biological versus technical replication
- The data obtained should be provided and sufficient information should be provided to indicate the number of independent biological and/or technical replicates
- If you encountered any outliers, you should describe how these were handled
- Criteria for exclusion/inclusion of data should be clearly stated
- High-throughput sequence data should be uploaded before submission, with a private link for reviewers provided (these are available from both GEO and ArrayExpress)

Please outline where this information can be found within the submission (e.g., sections or figure legends), or explain why this information doesn’t apply to your submission:

The number of replicates for each condition can be found in Figure 2 – supplement 1. This includes the number of mice used, the number of imaging sessions per condition, as well as the number of neurons recorded.

Only imaging sessions that meet our QC criteria (as described in de Vries et al., 2020, Nature Neuroscience) were included in the analysis shown in the manuscript. Examples of QC criteria that resulted in exclusion of data from the study include z-drift greater than 10um over the course of the 1-hour imaging session, signal saturation or bleaching, or low signal to noise.

**Statistical reporting**

- Statistical analysis methods should be described and justified
- Raw data should be presented in figures whenever informative to do so (typically when N per group is less than 10)
- For each experiment, you should identify the statistical tests used, exact values of N, definitions of center, methods of multiple test correction, and dispersion and precision measures (e.g., mean, median, SD, SEM, confidence intervals; and, for the major substantive results, a measure of effect size (e.g., Pearson's r, Cohen's d)
- Report exact p-values wherever possible alongside the summary statistics and 95% confidence intervals. These should be reported for all key questions and not only when the p-value is less than 0.05.

Please outline where this information can be found within the submission (e.g., sections or figure legends), or explain why this information doesn’t apply to your submission:

Description of the statistical tests used can be found in the ‘Statistics’ section of Materials and Methods. Exact p-values are reported are reported in cases where P was greater than 0.008 (the Bonferroni corrected alpha value given the number of comparisons in the study), otherwise significance of results is indicated as p<0.008 in the figure legends and throughout the text. Visualization methods including statistical measures (e.g. error bars show 95% confidence intervals) are described in the ‘Data Analysis’ section of Materials and Methods.

(For large datasets, or papers with a very large number of statistical tests, you may upload a single table file with tests, Ns, etc., with reference to sections in the manuscript.)

**Group allocation**

- Indicate how samples were allocated into experimental groups (in the case of clinical studies, please specify allocation to treatment method); if randomization was used, please also state if restricted randomization was applied
- Indicate if masking was used during group allocation, data collection and/or data analysis

Please outline where this information can be found within the submission (e.g., sections or figure legends), or explain why this information doesn’t apply to your submission:

In this study, experimental groups were determined by the transgenic mouse line that was used to label different cell classes. As such, no randomized or blinded group allocation was performed.

**Additional data files (“source data”)**

- We encourage you to upload relevant additional data files, such as numerical data that are represented as a graph in a figure, or as a summary table
- Where provided, these should be in the most useful format, and they can be uploaded as “Source data” files linked to a main figure or table
- Include model definition files including the full list of parameters used
- Include code used for data analysis (e.g., R, MatLab)
- Avoid stating that data files are “available upon request”

Please indicate the figures or tables for which source data files have been provided:

Source data will be made publicly available at the time of publication. Analysis code will be made available on GitHub.
